# Prevalence of Homologous Recombination Deficiency Among Patients With Germline *RAD51C/D* Breast or Ovarian Cancer

**DOI:** 10.1001/jamanetworkopen.2024.7811

**Published:** 2024-04-22

**Authors:** Sara Torres-Esquius, Alba Llop-Guevara, Sara Gutiérrez-Enríquez, Marcel Romey, Àlex Teulé, Gemma Llort, Ana Herrero, Pilar Sánchez-Henarejos, Anna Vallmajó, Santiago González-Santiago, Isabel Chirivella, Juana Maria Cano, Begoña Graña, Sara Simonetti, Isabela Díaz de Corcuera, Teresa Ramon y Cajal, Judit Sanz, Sara Serrano, Andrea Otero, Cristina Churruca, Ana Beatriz Sánchez-Heras, Sonia Servitja, Carmen Guillén-Ponce, Joan Brunet, Carsten Denkert, Violeta Serra, Judith Balmaña

**Affiliations:** 1 Hereditary Cancer Genetics Group, Vall d’Hebron Institute of Oncology, Barcelona, Spain; 2 Experimental Therapeutics Group, Vall d’Hebron Institute of Oncology, Barcelona, Spain; 3 Translational Medicine, DNA Damage Response Department, AstraZeneca, Barcelona, Spain; 4 Institute of Pathology, Universitätsklinikum Marburg, Marburg, Germany; 5 Hereditary Cancer Program, Catalan Institute of Oncology, Bellvitge Biomedical Research Institute (IDIBELL), Centro de Investigación Biomédica en Red de Cáncer (CIBERONC), Madrid, Spain; 6 Department of Medical Oncology, Hospital Universitari Parc Taulí, Sabadell, Spain; 7 Department of Medical Oncology, Hospital Miguel Servet de Zaragoza, Zaragoza, Spain; 8 Department of Medical Oncology, Clinical University Hospital Virgen Arrixaca, Murcia, Spain; 9 Genetic Counseling Unit, Arnau de Vilanova University Hospital, Lleida, Spain; 10 Department of Medical Oncology, Hospital San Pedro de Alcántara, Cáceres, Spain; 11 Cancer Genetic Counseling, Hospital Clínico Universitario de Valencia, Valencia, Spain; 12 Department of Medical Oncology, Hospital General Universitario de Ciudad Real, Ciudad Real, Spain; 13 Department of Medical Oncology, Xerencia de Xestión Integrada de A Coruña, Coruña, Spain; 14 Molecular Oncology Group, Vall d’Hebron Institute of Oncology, Barcelona, Spain; 15 Department of Medical Oncology, Hospital Universitario de Galdakao, Galdakao-Usansolo, Spain; 16 Department of Medical Oncology, Hospital de la Santa Creu i Sant Pau, Barcelona, Spain; 17 Unidad de Cáncer Familiar y Hereditario, Althaia Xarxa Assistencial Universitària de Manresa, Manresa, Spain; 18 Department of Medical Oncology, Institute of Oncology of Southern Catalonia (IOCS), Hospital Universitari Sant Joan de Reus, Reus, Spain; 19 Institute of Oncology and Molecular Medicine of Asturias (IMOMA) S. A., Oviedo, Spain; 20 Department of Medical Oncology, Hospital Universitario Donostia, San Sebastián, Gipuzkoa, Spain; 21 Cancer Genetic Counselling Unit, Medical Oncology Department, Hospital General Universitario de Elche, Elche, Spain; 22 Department of Medical Oncology, Hospital del Mar-CIBERONC, Barcelona, Spain; 23 Department of Medical Oncology, Hospital Universitario Ramón y Cajal (IRYCIS), Madrid, Spain; 24 Hereditary Cancer Program, Catalan Institute of Oncology, Girona, Spain; 25 Precision Oncology Group (OncoGIR-Pro), Institut d’Investigació Biomèdica de Girona (IDIBGI), Girona, Spain; 26 Medical Oncology Department, Hospital Universitari Vall d’Hebron, Barcelona, Spain

## Abstract

**Importance:**

*RAD51C* and *RAD51D* are involved in DNA repair by homologous recombination. Germline pathogenic variants (PVs) in these genes are associated with an increased risk of ovarian and breast cancer. Understanding the homologous recombination deficiency (HRD) status of tumors from patients with germline PVs in *RAD51C/D* could guide therapeutic decision-making and improve survival.

**Objective:**

To characterize the clinical and tumor characteristics of germline *RAD51C/D* PV carriers, including the evaluation of HRD status.

**Design, Setting, and Participants:**

This retrospective cohort study included 91 index patients plus 90 relatives carrying germline *RAD51C/D* PV (n = 181) in Spanish hospitals from January 1, 2014, to December 31, 2021. Genomic and functional HRD biomarkers were assessed in untreated breast and ovarian tumor samples (n = 45) from June 2022 to February 2023.

**Main Outcomes and Measures:**

Clinical and pathologic characteristics were assessed using descriptive statistics. Genomic HRD by genomic instability scores, functional HRD by RAD51, and gene-specific loss of heterozygosity were analyzed. Associations between HRD status and tumor subtype, age at diagnosis, and gene-specific loss of heterozygosity in *RAD51C/D* were investigated using logistic regression or the *t* test.

**Results:**

A total of 9507 index patients were reviewed, and 91 patients (1.0%) were found to carry a PV in *RAD51C/D*; 90 family members with a germline PV in *RAD51C/D* were also included. A total of 157 of carriers (86.7%) were women and 181 (55.8%) had received a diagnosis of cancer, mainly breast cancer or ovarian cancer. The most prevalent PVs were c.1026+5_1026+7del (11 of 56 [19.6%]) and c.709C>T (9 of 56 [16.1%]) in *RAD51C* and c.694C>T (20 of 35 [57.1%]) in *RAD51D*. In untreated breast cancer and ovarian cancer, the prevalence of functional and genomic HRD was 55.2% (16 of 29) and 61.1% (11 of 18) for *RAD51C*, respectively, and 66.7% (6 of 9) and 90.0% (9 of 10) for *RAD51D*. The concordance between HRD biomarkers was 91%. Tumors with the same PV displayed contrasting HRD status, and age at diagnosis did not correlate with the occurrence of HRD. All breast cancers retaining the wild-type allele were estrogen receptor positive and lacked HRD.

**Conclusions and Relevance:**

In this cohort study of germline *RAD51C/D* breast cancer and ovarian cancer, less than 70% of tumors displayed functional HRD, and half of those that did not display HRD were explained by retention of the wild-type allele, which was more frequent among estrogen receptor–positive breast cancers. Understanding which tumors are associated with *RAD51C/D* and HRD is key to identify patients who can benefit from targeted therapies, such as PARP (poly [adenosine diphosphate–ribose] polymerase) inhibitors.

## Key Points

**Question:**

What is the prevalence of homologous recombination deficiency (HRD) in tumors from patients with germline *RAD51C/D* breast and ovarian cancer?

**Findings:**

In this cohort study, the prevalence of HRD based on genomic and functional tumor biomarkers in germline *RAD51C/D* carriers was less than 70%. All estrogen receptor–positive breast cancers lacked HRD, in part associated with the retention of the wild-type allele in *RAD51C/D.*

**Meaning:**

These findings highlight the importance of HRD testing to guide therapeutic decision-making for patients with *RAD51C/D*-associated cancer.

## Teaser

This cohort study characterizes the clinical and tumor characteristics of germline *RAD51C/D* PV carriers, including the evaluation of homologous recombination deficiency status.

## Introduction

RAD51C and RAD51D are RAD51 paralogs involved in the homologous recombination repair (HRR) of double-stranded DNA breaks. Together with other RAD51 family members, they form protein complexes (BCDX2 and CX3) that act within the BRCA1/2-dependent HRR pathway and contribute to genomic stability. Germline pathogenic variants (PVs) in *RAD51C* (OMIM 602774) and *RAD51D* (OMIM 602954) (*RAD51C/D*) are expected to cause homologous recombination deficiency (HRD) and genomic instability when there is biallelic inactivation, mainly through gene-specific loss of heterozygosity (gsLOH). As a result, germline PV carriers have an increased risk of ovarian cancer and breast cancer, particularly estrogen receptor (ER)–negative breast cancer.^1,2,3,4,5,6,7,8^ In this regard, germline PVs in *RAD51C*/*D* are found in 0.3% of patients with breast cancer and 1% of patients with ovarian cancer.^1,2,9,10,11^

Current methods to assess HRD fall into 3 categories: HRR gene panel sequencing, genomic scars and signatures, and functional assays.^12^ Selection of patients for treatment with a poly (adenosine diphosphate–ribose) polymerase (PARP) inhibitor is currently based on germline *BRCA1/2* (*BRCA1*, OMIM 113705; *BRCA2*, OMIM 600185) mutation status for breast cancer or platinum sensitivity, *BRCA1/2* alteration, or genomic HRD for ovarian cancer.^12^ Regarding functional assays of HRD, studies have shown that the RAD51 assay can effectively identify tumors with HRD that are sensitive to platinum and PARP inhibitors, albeit this functional assay has not yet been validated for treatment selection.^13,14,15,16,17,18,19,20^

The prevalence of genomic HRD in tumors of *RAD51C/D* PV carriers has mainly been investigated within large cohorts of pan-cancer HRD analysis.^21^ In a small sample, Li et al^22^ showed that 7 of 9 cases of *RAD51C*-associated breast cancer (77.8%) harbored genomic HRD based on a high genomic instability score (GIS) and concomitant gsLOH. In ARIEL2, Swisher et al^23,24^ showed that mutations in *RAD51C/D* were associated with genomic HRD (based on high genomic LOH) and response to the PARP inhibitor rucaparib in 5 of 7 patients (71.4%) with relapsed high-grade ovarian cancer, reaching a median progression-free survival similar to patients with mutated *BRCA1/2*. Similarly, one study showed a high sensitivity to DNA-damaging chemotherapy in a patient with breast cancer with a *RAD51D* germline PV and functional HRD.^25^ Overall, prior clinical trials in breast cancer or ovarian cancer have analyzed the efficacy of platinums and PARP inhibitors for patients with germline *RAD51C/D* PVs observing a wide range of treatment responses.^26,27^ Some studies have reported the presence of gsLOH^26^ but lack of concordance with HRD by GIS, and others do not report biallelic inactivation or HRD status.^28^ In summary, prior evidence highlights the necessity of knowing the HRD functional status of *RAD51C/D* germline carriers with cancer to determine whether they might benefit from targeted therapeutic management. We aimed to perform a comprehensive molecular analysis of a large cohort of patients with *RAD51C/D* untreated primary breast cancer and ovarian cancer to describe the prevalence of HRD by different biomarkers and investigate the role of the germline alterations in tumorigenesis.

## Methods

### Study Population

Between January 1, 2014, and December 31, 2021, 9507 individuals from 18 hereditary cancer units across Spain underwent germline genetic testing for breast cancer and/or ovarian cancer predisposition. This retrospective cohort study included women and men with *RAD51C* or *RAD51D* germline PVs, as well as family members carrying these variants. We did not check for sample size using a power analysis because our study included all patients older than 18 years tested routinely in screening programs. All participants provided written informed consent before study entry and were codified by their respective center. This study followed the Strengthening the Reporting of Observational Studies in Epidemiology (STROBE) reporting guideline and was reviewed and approved by, and conducted according to, the ethical standards of the Vall d’Hebron Hospital Ethics Committee and all institutional review boards of the participating centers (Catalan Institute of Oncology, Hospital Universitari Parc Taulí, Hospital Miguel Servet de Zaragoza, Clinical University Hospital Virgen Arrixaca, Arnau de Vilanova University Hospital, Hospital San Pedro de Alcántara, Clínico Universitario de Valencia, Hospital General Universitario de Ciudad Real, Xerencia de Xestión Integrada de A Coruña, Hospital Universitario de Galdakao, Hospital de la Santa Creu i Sant Pau, Althaia Xarxa Assistencial Universitària de Manresa, Institute of Oncology of Southern Catalonia, Hospital Universitari Sant Joan de Reus, Institute of Oncology and Molecular Medicine of Asturias [IMOMA], Hospital Universitario Donostia, Hospital General Universitario de Elche, Hospital del Mar, and Hospital Universitario Ramón y Cajal). In addition, 103 primary breast cancer samples from patients carrying a germline PV in *BRCA1* (n = 47), *BRCA2* (n = 36), and *PALB2* (OMIM 610355) (n = 20) from the Vall d’Hebron Hereditary Cancer Unit were used as controls for comparison with the germline *RAD51C/D* tumor samples.

Variants were classified by each independent laboratory and subsequently reviewed by the central laboratory according to the American College of Medical Genetics and Genomics guidelines.^29^ The carrier frequency for *RAD51C/D* PVs was calculated as the number of index patients with a PV in *RAD51C/D* divided by the total number of index patients tested for *RAD51C/D*.

### HRR Assays

Formalin-fixed, paraffin-embedded (FFPE) tumor samples were requested from the participating centers in 2022. HRD analyses were performed from June 2022 to February 2023.

#### RAD51 Immunofluorescence Test

To evaluate the functional HRR status with the RAD51 test, FFPE whole tumor sections (3 μm) from early untreated breast cancer and ovarian cancer were used to detect RAD51 foci (as a functional readout of HRD), γH2AX foci (as a biomarker of double strand DNA breaks), and BRCA1. Each biomarker was counterstained with geminin (as a marker of S/G2 cell cycle phase) and DAPI (4′,6-diamidino-2-phenylindole). Commercially available primary and secondary antibodies were used as per the protocol in a previous study.^17^ The scoring was performed blindly onto life images using a ×60-immersion oil objective in a Nikon Ti2-Eclipse microscope. At least 40 geminin-positive cells were analyzed per sample, and γH2AX scoring was used as a quality check to ensure the presence of endogenous DNA damage to evaluate HRR functionality (cutoff: 25% geminin-positive cells with γH2AX foci). RAD51 and BRCA1 scores were considered low or high based on the predefined cutoff of 10% geminin-positive cells with 5 or more RAD51 or BRCA1 nuclear foci or cells.^13,15,16,17^ Functional HRD was defined by low RAD51 scores (≤10%), and functional homologous recombination proficiency (HRP) by high RAD51 scores (>10%).

#### Gene Sequencing and Genomic Instability

To assess genetic or genomic HRD, the Myriad myChoice HRD Plus CDx assay was performed at Philipps-Universität Marburg, as described in previous studies.^30,31,32^ Tumor DNA was isolated from FFPE samples and used for targeted multiplex polymerase chain reaction amplification and library construction. Next-generation sequencing (Illumina) was conducted to screen tumor mutations of *BRCA1* and *BRCA2* and 13 additional genes relevant to DNA repair (*ATM* [OMIM 607585], *BARD1* [OMIM 601593], *BRIP1* [OMIM 605882], *CDK12* [OMIM 615514], *CHEK1* [OMIM 603078], *CHEK2* [OMIM 604373], *FANCL* [OMIM 608111], *PALB2* [OMIM 610335], *PPP2R2A* [OMIM 604941], *RAD51B* [OMIM 602948], *RAD51C* [OMIM 602774], *RAD51D* [OMIM 602954], and *RAD54L* (OMIM 603615]). A standardized bioinformatic analysis was used to determine the GIS based on loss of heterozygosity, telomeric allelic imbalance, and large-scale state transitions.^33^ Genomic HRD was defined as a GIS of 42 or higher. To estimate the gsLOH status of the *RAD51C/D* loci and other HRR genes, the computationally most likely allele-specific copy number at each single-nucleotide variation location was analyzed.

### Statistical Analysis

A descriptive analysis was performed to describe the study population. Continuous variables were expressed as median (IQR) values, and categorical variables were expressed as absolute values and percentages. The Cohen κ coefficient was used to analyze the concordance between HRD assays. The association among HRD, gsLOH, specific tumor subtype, and age at diagnosis was evaluated using the *t* test, univariate logistic regression, or univariate logistic regression with the Firth bias reduction method (to solve the problem of perfect separation). All *P* values were from 1-sided tests and results were deemed statistically significant at *P* < .05, and 95% CIs were reported. Analyses were performed with R statistical software, version 4.1.1 (R Project for Statistical Computing).

## Results

### Patient Characteristics

Genetic susceptibility to breast and/or ovarian cancer was assessed for 9507 index patients. Among them, 91 had a PV in *RAD51C/D*. Furthermore, the study encompassed 90 family members with a germline PV in *RAD51C/D*. In total, 181 individuals were included, with 113 carrying *RAD51C* PVs and 68 carrying *RAD51D* PVs (Table 1). A total of 157 carriers (86.7%) were women and 181 (55.8%) had received a diagnosis of cancer, primarily breast cancer or ovarian cancer. Additional details of the study population are presented in Table 1.

**Table 1.** Study Population

Characteristic	No. (%) (N = 181)
Sex	
Female	157 (86.7)
Male	24 (13.3)
Type of gene	
* RAD51C*	113 (62.4)
* RAD51D*	68 (37.6)
Type of cancer	
Breast cancer	34 (18.8)
Ovarian cancer	45 (24.9)
Breast cancer and ovarian cancer	7 (3.9)
Multiple primary breast cancer	5 (2.8)
Other non–breast cancer or non–ovarian cancer	10 (5.5)
No cancer	80 (44.2)
Index cases with pathogenic variant (n = 91)	
Female	88 (96.7)
Male	3 (3.3)
* RAD51C*	56 (61.5)
* RAD51D*	35 (38.5)
Family history	
Non–breast cancer or non–ovarian cancer family history	45 (49.5)
Only breast cancer family history	27 (29.7)
Only ovarian cancer family history	14 (15.4)
Breast cancer and ovarian cancer family history	4 (4.4)
Unknown family history	1 (1.1)
Type of cancer	
Breast cancer	34 (37.4)
Age at diagnosis breast cancer, median (IQR), y	39 (36-49)
Ovarian cancer	41 (45.1)
Age at diagnosis ovarian cancer, median (IQR), y	61 (56-66)
Breast cancer and ovarian cancer	7 (7.7)
Other non–breast cancer or non–ovarian cancer	4 (4.4)
No cancer	5 (5.5)

### Prevalence of Pathogenic and Likely Pathogenic *RAD51C/D* Variants

Overall, 1.0% of individuals (91 of 9507) were found to have a PV in *RAD51C/D*, with 56 (0.6%) carrying *RAD51C* PV and 35 (0.4%) carrying *RAD51D* PV (Table 1). Among the 56 *RAD51C* carriers, we identified 22 unique variants. Two variants, c.1026+5_1026+7del and c.709C>T, were particularly prevalent in the cohort, with 19.6% (11 of 56) unrelated individuals carrying c.1026+5_1026+7del and 16.1% (9 of 56) unrelated patients carrying c.709C>T. Among the 35 *RAD51D* carriers, we identified 8 unique variants, with 1 variant, c.694C>T, being present in 57.1% of unrelated individuals (20 of 35) (eTables 1 and 2 in Supplement 1).

### Clinical Characteristics of *RAD51C/D*-Associated Breast and Ovarian Cancers

The clinical characteristics of patients with *RAD51C/D* breast cancer are summarized in Table 2. Of 113 patients carrying *RAD51C*, 32 (28.3%) had received a diagnosis of breast cancer, and 4 women had a second primary breast cancer. The median age at diagnosis was 43 years (IQR, 39-64 years). Most tumors were invasive ductal carcinoma (32 of 36 [88.9%]) and were diagnosed at anatomic stages I or II (26 of 36 [72.2%]). With respect to hormone receptor status, 52.8% (19 of 36) had ER-negative tumors, and 41.7% (15 of 35) had triple-negative breast cancer. Among 68 *RAD51D* carriers, 20.6% (14 of 68) had received a diagnosis of breast cancer, and 1 woman had a second primary breast cancer. The median age at diagnosis was 38 years (IQR, 35-41 years). All tumors but 1 were invasive ductal carcinoma, and 66.7% (10 of 15) were diagnosed at stages I or II. The distribution of hormonal receptor status was also similar between the 2 genes, with 53.3% (8 of 15) of *RAD51D* breast cancers being ER negative and 46.7% (7 of 15) being triple-negative breast cancers.

**Table 2.** Characteristics of *RAD51C/D*-Associated Breast Cancers

Characteristic	No./total No. (%)	
	*RAD51C* (36 cancers from 32 patients)	*RAD51D* (15 cancers from 14 patients)
Age at diagnosis, median (IQR), y^a^	43 (39-64)	38 (35-41)
Multiple breast cancer	4/32 (12.5)	1/14 (7.1)
Histologic characteristics		
Invasive ductal carcinoma	32/36 (88.9)	14/15 (93.3)
Invasive lobular carcinoma	3/36 (8.3)	NA
NA	1/36 (2.8)	1/15 (6.7)
Receptor status		
ER positive, PR positive, ERBB2 negative	10/36 (27.8)	2/15 (13.3)
ER positive, PR, negative, ERBB2 negative	4/36 (11.1)	2/15 (13.3)
ER negative, PR positive, ERBB2 negative	2/36 (5.6)	NA
ER negative, PR negative, ERBB2 positive	2/36 (5.6)	NA
ER negative, PR negative, ERBB2 status NA	NA	1/15 (6.7)
Triple negative	15/36 (41.7)	7/15 (46.7)
ER positive, other unknown	1/36 (2.8)	NA
NA	2/36 (5.6)	3/15 (20.0)
Clinical stage		
IA	11/36 (30.6)	1/15 (6.7)
IIA	11/36 (30.6)	5/15 (33.3)
IIB	4/36 (11.1)	4/15 (26.7)
IIIA	2/36 (5.6)	NA
IIIB	1/36 (2.8)	1/15 (6.7)
IV	3/36 (8.3)	NA
NA	4/36 (11.1)	4/15 (26.7)

Abbreviations: ER, estrogen receptor; NA, not available; PR, progesterone receptior.

^a^ Only first breast cancer diagnosis.

The characteristics of *RAD51C/D*-associated ovarian cancer are summarized in Table 3. Among women carrying *RAD51C* alterations, 27.4% (31 of 113) had received a diagnosis of ovarian cancer. The median age at diagnosis was 63 years (IQR, 60-68 years), with 5 patients diagnosed before 50 years of age. Most (83.9% [16 of 31]) had serous adenocarcinoma, and 71.0% (22 of 31) received a diagnosis at an advanced stage (International Federation of Gynecology and Obstetrics [FIGO] stage III or IV). For *RAD51D*, 30.9% of women (21 of 68) had received a diagnosis of ovarian cancer. The median age was 59 years (IQR, 54-67 years), with 5 patients diagnosed before 50 years of age. Most tumors (90.5% [19 of 21]) were serous adenocarcinomas, and 81.0% (17 of 21) were diagnosed at an advanced stage (FIGO stage III or IV). All serous carcinomas were high grade.

**Table 3.** Characteristics of *RAD51C/D*-Associated Ovarian Cancers

Characteristic	No. (%)	
	*RAD51C* (31 cancers from 31 patients)	*RAD51D* (21 cancers from 21 patients)
Age at diagnosis, median (IQR), y	63 (60-68)	59 (54-67)
Histology		
Serous carcinoma	26 (83.9)	19 (90.5)
Endometriod carcinoma	2 (6.5)	NA
Carcinosarcoma	1 (3.2)	NA
Mucinous	NA	2 (9.5)
NA	2 (6.5)	NA
FIGO staging		
I	5 (16.1)	1 (4.8)
II	3 (9.7)	2 (9.5)
III	14 (45.2)	10 (47.6)
IV	8 (25.8)	7 (33.3)
NA	1 (3.2)	1 (4.8)

Abbreviations: FIGO, International Federation of Gynecology and Obstetrics; NA, not applicable.

In summary, *RAD51C/D* carriers with breast cancer had a median age at diagnosis of 39 years (IQR, 36-49 years) and were enriched for ER-negative phenotype. Among patients with ovarian cancer, 19.6% received a diagnosis before 50 years of age, and most had high-grade serous ovarian cancer in an advanced clinical stage.

### Assessment of the HRD Status

**Figure 1.**
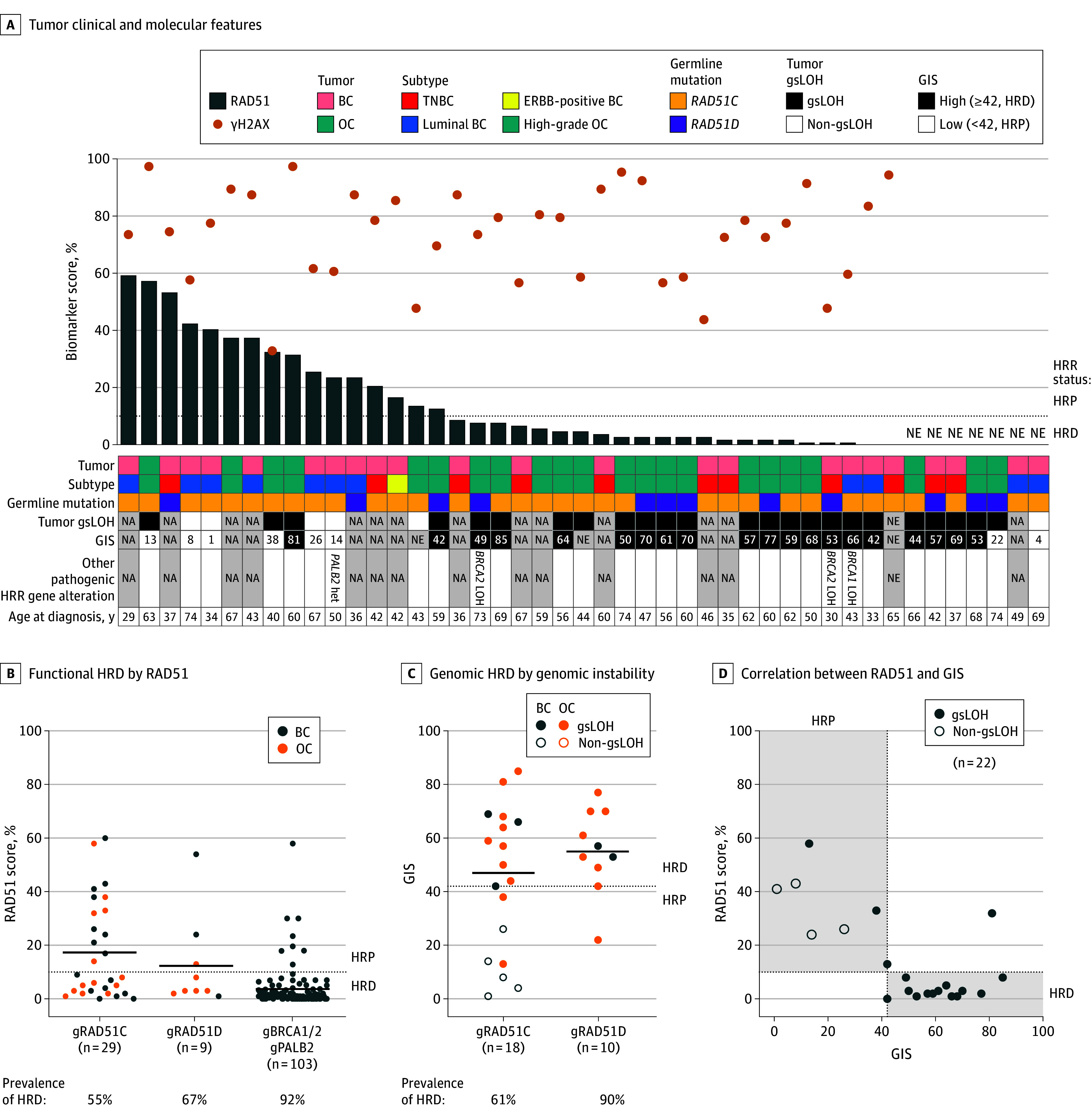
Functional and Genomic Homologous Recombination Deficiency (HRD) A, Summary of clinical and molecular features for the 45 breast cancer (BC) or ovarian cancer (OC) tumors analyzed. Waterfall with the RAD51 scores (bars) and yH2AX scores (dots) for each sample. The table indicates the type of each tumor, gene mutated, gene-specific loss of heterozygosity (gsLOH) status, genomic instability score (GIS), and age at diagnosis. B, Functional HRD by RAD51 in hereditary cancers. The RAD51 scores of 141 tumor samples from patients with BC or OC with germline pathogenic variants in *RAD51C*, *RAD51D*, *BRCA1*, *BRCA2*, or *PALB2* are shown. C, Genomic HRD by genomic instability. The GIS of 28 tumor samples from patients with BC or OC with germline pathogenic variants in *RAD51C* or *RAD51D* are shown. The gsLOH status in *RAD51C/D* is also shown. D, Correlation between RAD51 and GIS, showing a 91% concordance. Each dot represents 1 tumor per patient, the horizontal black lines indicate the mean of each group, and the horizontal dotted lines indicate the predefined threshold of the RAD51 test (10%) or GIS (42) to discriminate HRD vs homologous recombination proficiency (HRP) status. Gray shaded areas in panel D represent concordant HRD or HRP status by both tests. Het indicates heterozygous; HRR, homologous recombination repair; NA, not available; NE, not evaluable; and TNBC, triple-negative breast cancer.

Of 181 patients, 98 had breast cancer and/or ovarian cancer. From those, we obtained 45 untreated FFPE tumor samples (23 breast cancer and 22 ovarian cancer) to evaluate the HRD status (eFigure 1 in Supplement 1). Two samples with insufficient tumor content and 15 samples with insufficient tissue material or DNA were excluded from the functional and genetic or genomic HRD analyses, respectively. The RAD51 foci test was successful in 88.4% of samples (38 of 43). Five samples were nonevaluable due to poor tissue quality. The Myriad myChoice HRD test was successful in 93.3% of samples (28 of 30). Two samples were nonevaluable for GIS due to poor DNA quality, although they were evaluable for HRR gene mutation calling and gsLOH status (eFigure 1 in Supplement 1). All germline PVs in *RAD51C* and *RAD51D* were identified in the respective tumors. Panel sequencing of HRR-related genes additionally identified 1 tumor with a likely *BRCA1* PV with gsLOH, 2 with *BRCA2* PVs with gsLOH, and 1 tumor with a PV in *PALB2* without gsLOH (Figure 1A). All germline *RAD51C/D* tumors had high levels of nuclear BRCA1 foci, which excluded potential concomitant epigenetic silencing of *BRCA1* as the origin of HRD,^14^ except for 1 *RAD51C* carrier with low levels of BRCA1 foci likely due to a concomitant tumor *BRCA1* PV (eFigure 2 in Supplement 1). In summary, 13.3% of tumors (4 of 30) from patients with germline *RAD51C/D* PVs concomitantly carried mutations in other HRR genes, and none showed epigenetic silencing of BRCA1.

### Prevalence of Functional and Genomic HRD in Hereditary *RAD51C/D*-Associated Cancers

The levels of endogenous DNA damage in primary untreated *RAD51C/D* tumors were high (mean score, 74% yH2AX; eFigure 2 in Supplement 1). The prevalence of functional HRD by RAD51 was 55.2% in germline *RAD51C* tumors (16 of 29) and 66.7% in germline *RAD51D* tumors (6 of 9) (Figure 1B). Overall, functional HRD was more prevalent in ovarian cancer (68.4% [13 of 19]) than in breast cancer (47.4% [9 of 19]). As a comparison, we included the analysis of RAD51 foci in primary tumor samples from patients with untreated breast cancer with germline PVs in *BRCA1*, *BRCA2*, and *PALB2*, which showed a high prevalence of HRD (92.2% [95 of 103]), as expected (Figure 1B). We next studied whether the functional HRD status of *RAD51C/D* tumors varied across PVs. Different tumors with the same PV showed variable HRD status, regardless of cancer type (eFigure 3 in Supplement 1). In particular, functional HRD values varied in tumors with the following PVs in *RAD51C*: deletion of exons 4 to 9, c.705+1G>A, c.709C>T, c.965+5G>A, c.979_989dup, and c.1026+5_1026+7del; and in *RAD51D*, c.94_95del and c.694C>T.

The prevalence of genomic HRD by GIS was 61.1% in germline *RAD51C* tumors (11 of 18) and 90.0% in germline *RAD51D* tumors (9 of 10) (Figure 1C). Similar to RAD51, HRD was more prevalent in ovarian cancer (83.3% [15 of 18]) than in breast cancer (50.0% [5 of 10]). Additional analysis of gsLOH status revealed that 80.0% of the studied tumors (24 of 30) had gsLOH in *RAD51C/D*. Moreover, 62.5% of tumors (5 of 8) with low GIS retained the wild-type allele (non-gsLOH), which could explain the lack of an HRD profile (Figure 1A and C).

The concordance between genomic and functional HRD was 91% (Cohen κ = 0.8 [95% CI, 0.5-1.0]; *P* < .001) (Figure 1D; eFigure 4 in Supplement 1), with 63.6% of tumors (14 of 22) harboring HRD by both techniques and 27.3% (6 of 22) showing HRP. The concordance between gsLOH and GIS was 76%, and between gsLOH and RAD51, it was 83% (eFigure 4 in Supplement 1). Tumors with non-gsLOH in *RAD51C* showed HRP, with RAD51 foci formation and low GIS. Discordancy was observed in 1 ovarian cancer case with a germline *RAD51D* PV, which showed borderline results for both genomic instability and RAD51 foci (GIS of 42 and 13% RAD51). The other case was a surgical ovarian cancer specimen with a germline *RAD51C* PV, showing HRD by GIS (81) and HRP by RAD51 (32%). Overall, functional and genomic HRD were highly concordant and ranged between 55% and 90% depending on the gene and type of tumor.

### Association of HRD With Age and Cancer Subtype

**Figure 2.**
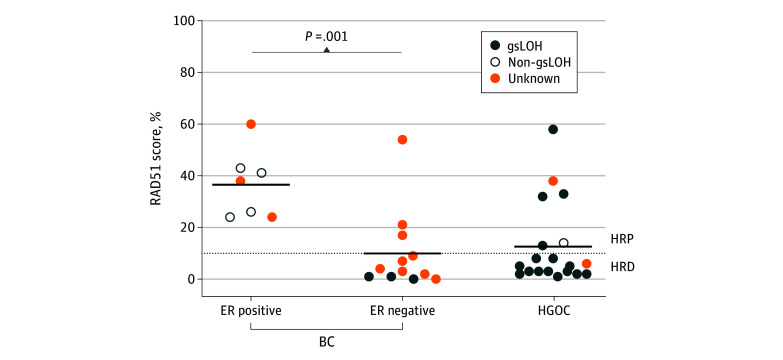
Distribution of Functional Homologous Recombination Deficiency (HRD) Across Tumor Subtypes RAD51 scores in estrogen receptor (ER)–positive breast cancer (BC), ER-negative BC, and high-grade ovarian cancer (HGOC) samples and gene-specific loss of heterozygosity (gsLOH) status. The horizontal black lines indicate mean values. HRP indicates homologous recombination proficiency.

We investigated whether lack of HRD was more common in patients with an older age (>50 years) at onset, suggesting that their tumors were of sporadic vs hereditary origin. However, we found no significant association between age at diagnosis and HRD by RAD51 or gsLOH (eFigure 5A-C in Supplement 1). Finally, we stratified the results by cancer subtypes, namely ER-positive breast cancer, ER-negative breast cancer, and high-grade ovarian cancer, as all ovarian cancer samples analyzed were of high grade (Figure 2; eFigure 5D-E in Supplement 1). One of the RAD51 high ER-negative breast cancer cases was an ERBB2-positive tumor (Figure 1A). Estrogen receptor–positive breast cancer had a higher prevalence of HRP and concomitant non-gsLOH compared with ER-negative breast cancer and high-grade ovarian cancer (Figure 2B).

## Discussion

To our knowledge, it is currently unclear whether patients with germline PVs in *RAD51C/D* can benefit from DNA damage repair–targeted agents, such as PARP inhibitors. Homologous recombination deficiency, mainly occurring in mutated *BRCA1/2* tumors, has been shown to be a potent biomarker of PARP inhibitor response. Therefore, we aimed to investigate the frequency of HRD among patients with cancer with germline PVs in *RAD51C/D*. Unexpectedly, we observed that the incidence of HRD in germline *RAD51C/D* was lower than in germline *BRCA1/2* or *PALB2*, especially among patients with ER-positive breast cancer.

In this study of 9507 index patients, the prevalence of an *RAD51C/D* PV was 1.0%, slightly higher than in population-based studies.^1,2^ Almost half of the index patients had no family history of breast cancer or ovarian cancer, compatible with the moderate cancer risk associated with these gene alterations.^3^ One variant (*RAD51D* c.694C>T) was highly prevalent in our cohort (57.1%), and although it had previously been reported elsewhere,^34^ its high frequency may suggest a founder origin. Within this cohort, we further characterized 113 individuals who carried a germline *RAD51C* PV and 68 individuals who carried a germline *RAD51D* PV. Half the individuals had received a diagnosis of cancer, primarily breast or ovarian cancer. The clinical characteristics of breast cancer or ovarian cancer were similar between carriers of *RAD51C* and carriers of *RAD51D*. Breast cancer cases were enriched for ER-negative phenotype (52.8%), an aggressive tumor type lacking targeted therapies apart from the use of PARP inhibitors for patients with germline *BRCA1/2* PVs. A total of 19.6% of patients with ovarian cancer received a diagnosis before 50 years of age, the majority at an advanced disease stage, which highlights the importance of preventive oophorectomy for female carriers of *RAD51C/D*.

The incidence of HRD in germline *RAD51C/D* was lower than in germline *BRCA1/2* or *PALB2*, especially among breast cancer samples. We investigated the potential explanation for the lower HRD frequency, including the type of mutation, age at diagnosis, gsLOH, or ER status. Different tumors with the same PV displayed contrasting HRD statuses, indicating no correlation between the PV type and HRD. Regarding age, we did not find any correlation between an earlier age at onset and a higher occurrence of HRD. The majority of ovarian cancers showed HRD associated with gsLOH, as previously reported^35,36^ and like ER-negative breast cancer. We found that all ER-positive breast cancer cases were HRP by RAD51 foci and lacked gsLOH. This finding is consistent with pan-cancer studies reporting moderate rates of biallelic inactivation among *RAD51C/D* cases compared with high rates in *BRCA1*/*2*.^37^ This finding also suggests that ER-positive breast cancer in patients with germline PVs in *RAD51C/D* might, in fact, be sporadic tumors. Similarly, Li et al^22^ found that 2 ER-positive cases out of 9 cases of breast cancer retained heterozygosity across the *RAD51C* locus and were the only cases of breast cancer that did not exhibit HRD. Our findings suggest that germline *RAD51C/D* PV is not associated with the tumorigenesis of ER-positive breast cancer, consistent with epidemiologic data showing that germline *RAD51C/D* PV carriers have a higher risk of developing ER-negative breast cancer.^1,2^

### Strengths and Limitations

A strength of the present work is the amount of *RAD51C/D* primary tumors that have been fully characterized for HRD status by genomic tests and the RAD51 functional test. There was a high level of agreement between GIS and RAD51 foci (91%), supporting prior data.^17^ There were only 2 discordant cases, both ovarian cancer. The first showed borderline values for both biomarkers, and the second was heterogeneous with subclones showing HRP by RAD51 despite an overall genomic HRD profile. The incidence of HRD in germline *RAD51C/D* was lower than in germline *BRCA1/2* or *PALB2*, especially among breast cancer samples.

The main limitation of this study is that all HRD biomarkers were not assessed in all samples mainly due to limited sample availability and quality. It remains to be further investigated how *RAD51C/D* germline mutation carriers respond to targeted therapies according to their HRD status, especially in ER-positive breast cancer, and the effect of RAD51C promoter methylation on HRD status and treatment response.^38^

## Conclusions

In this cohort study of germline *RAD51C/D* breast cancer or ovarian cancer, less than 70% of tumors displayed functional HRD, and half of those that did not display HRD could be explained by retention of the wild-type allele, which was more frequent among patients with ER-positive breast cancer. Therefore, it is key to investigate the molecular basis of these tumors to identify patients who might show HRD and would likely benefit from targeted therapies, such as PARP inhibitors.

## Notes

Supplement 1. **eTable 1.** Unique Pathogenic Variants in RAD51C (n=56) **eTable 2.** Unique Pathogenic Variants in RAD51D (n=35) **eFigure 1.** CONSORT Diagram **eFigure 2.** Analysis of Functional HRD Biomarkers by Immunofluorescence **eFigure 3.** Distribution of Functional HRD Across Tumors With Pathogenic Variants in RAD51C/D **eFigure 4.** Concordance Between HRD Tests: Functional HRD by RAD51, Genomic HRD by GIS and RAD51C/D Gene-Specific LOH **eFigure 5.** Comparison of HRR/gsLOH Status With Age at Diagnosis and Cancer Subtype

Supplement 2.
Data Sharing Statement

## References

[1] Dorling L, Carvalho S, Allen J, ; Breast Cancer Association Consortium. Breast cancer risk genes—association analysis in more than 113,000 women. N Engl J Med. 2021;384(5):428-439. doi:10.1056/NEJMoa1913948 33471991 PMC7611105

[2] Hu C, Hart SN, Gnanaolivu R, . A population-based study of genes previously implicated in breast cancer. N Engl J Med. 2021;384(5):440-451. doi:10.1056/NEJMoa2005936 33471974 PMC8127622

[3] Yang X, Song H, Leslie G, . Ovarian and Breast Cancer Risks Associated With Pathogenic Variants in RAD51C and RAD51D. J Natl Cancer Inst. 2020;112(12):1242-1250. doi:10.1093/jnci/djaa030 32107557 PMC7735771

[4] Song H, Dicks E, Ramus SJ, . Contribution of germline mutations in the *RAD51B*, *RAD51C*, and *RAD51D* genes to ovarian cancer in the population. J Clin Oncol. 2015;33(26):2901-2907. doi:10.1200/JCO.2015.61.2408 26261251 PMC4554751

[5] Shimelis H, LaDuca H, Hu C, . Triple-negative breast cancer risk genes identified by multigene hereditary cancer panel testing. J Natl Cancer Inst. 2018;110(8):855-862. doi:10.1093/jnci/djy106 30099541 PMC6093350

[6] Couch FJ, Hart SN, Sharma P, . Inherited mutations in 17 breast cancer susceptibility genes among a large triple-negative breast cancer cohort unselected for family history of breast cancer. J Clin Oncol. 2015;33(4):304-311. doi:10.1200/JCO.2014.57.1414 25452441 PMC4302212

[7] Couch FJ, Shimelis H, Hu C, . Associations between cancer predisposition testing panel genes and breast cancer. JAMA Oncol. 2017;3(9):1190-1196. doi:10.1001/jamaoncol.2017.0424 28418444 PMC5599323

[8] Greenhough LA, Liang CC, Belan O, . Structure and function of the RAD51B-RAD51C-RAD51D-XRCC2 tumour suppressor. Nature. 2023;619(7970):650-657. doi:10.1038/s41586-023-06179-1 37344587 PMC7614784

[9] Suszynska M, Ratajska M, Kozlowski P. *BRIP1*, *RAD51C*, and *RAD51D* mutations are associated with high susceptibility to ovarian cancer: mutation prevalence and precise risk estimates based on a pooled analysis of ~30,000 cases. J Ovarian Res. 2020;13(1):50. doi:10.1186/s13048-020-00654-3 32359370 PMC7196220

[10] Blanco A, Gutiérrez-Enríquez S, Santamariña M, . *RAD51C* germline mutations found in Spanish site-specific breast cancer and breast-ovarian cancer families. Breast Cancer Res Treat. 2014;147(1):133-143. doi:10.1007/s10549-014-3078-4 25086635

[11] Gutiérrez-Enríquez S, Bonache S, de Garibay GR, . About 1% of the breast and ovarian Spanish families testing negative for *BRCA1* and *BRCA2* are carriers of *RAD51D* pathogenic variants. Int J Cancer. 2014;134(9):2088-2097. doi:10.1002/ijc.28540 24130102

[12] Miller RE, Leary A, Scott CL, . ESMO recommendations on predictive biomarker testing for homologous recombination deficiency and PARP inhibitor benefit in ovarian cancer. Ann Oncol. 2020;31(12):1606-1622. doi:10.1016/j.annonc.2020.08.2102 33004253

[13] Cruz C, Castroviejo-Bermejo M, Gutiérrez-Enríquez S, . RAD51 foci as a functional biomarker of homologous recombination repair and PARP inhibitor resistance in germline BRCA-mutated breast cancer. Ann Oncol. 2018;29(5):1203-1210. doi:10.1093/annonc/mdy099 29635390 PMC5961353

[14] Castroviejo-Bermejo M, Cruz C, Llop-Guevara A, . A RAD51 assay feasible in routine tumor samples calls PARP inhibitor response beyond BRCA mutation. EMBO Mol Med. 2018;10(12):e9172. doi:10.15252/emmm.201809172 30377213 PMC6284440

[15] Pellegrino B, Herencia-Ropero A, Llop-Guevara A, . Preclinical in vivo validation of the RAD51 test for identification of homologous recombination-deficient tumors and patient stratification. Cancer Res. 2022;82(8):1646-1657. doi:10.1158/0008-5472.CAN-21-2409 35425960 PMC7612637

[16] Carreira S, Porta N, Arce-Gallego S, . Biomarkers associating with PARP inhibitor benefit in prostate cancer in the TOPARP-B trial. Cancer Discov. 2021;11(11):2812-2827. doi:10.1158/2159-8290.CD-21-0007 34045297 PMC9414325

[17] Llop-Guevara A, Loibl S, Villacampa G, . Association of RAD51 with homologous recombination deficiency (HRD) and clinical outcomes in untreated triple-negative breast cancer (TNBC): analysis of the GeparSixto randomized clinical trial. Ann Oncol. 2021;32(12):1590-1596. doi:10.1016/j.annonc.2021.09.003 34520831

[18] Blanc-Durand F, Yaniz-Galende E, Llop-Guevara A, . A RAD51 functional assay as a candidate test for homologous recombination deficiency in ovarian cancer. Gynecol Oncol. 2023;171:106-113. doi:10.1016/j.ygyno.2023.01.026 36868112

[19] Compadre AJ, van Biljon LN, Valentine MC, . RAD51 foci as a biomarker predictive of platinum chemotherapy response in ovarian cancer. Clin Cancer Res. 2023;29(13):2466-2479. doi:10.1158/1078-0432.CCR-22-3335 37097615 PMC10320470

[20] Pikkusaari S, Tumiati M, Virtanen A, . Functional homologous recombination assay on FFPE specimens of advanced high-grade serous ovarian cancer predicts clinical outcomes. Clin Cancer Res. 2023;29(16):3110-3123. doi:10.1158/1078-0432.CCR-22-3156 36805632 PMC10425726

[21] Nguyen L, Martens JWM, Van Hoeck A, Cuppen E. Pan-cancer landscape of homologous recombination deficiency. Nat Commun. 2020;11(1):5584. doi:10.1038/s41467-020-19406-4 33149131 PMC7643118

[22] Li N, McInerny S, Zethoven M, . Combined tumor sequencing and case-control analyses of *RAD51C* in breast cancer. J Natl Cancer Inst. 2019;111(12):1332-1338. doi:10.1093/jnci/djz045 30949688 PMC6910168

[23] Swisher EM, Kwan TT, Oza AM, . Molecular and clinical determinants of response and resistance to rucaparib for recurrent ovarian cancer treatment in ARIEL2 (parts 1 and 2). Nat Commun. 2021;12(1):2486. doi:10.1038/s41467-021-22582-6 33941784 PMC8093258

[24] Swisher EM, Lin KK, Oza AM, . Rucaparib in relapsed, platinum-sensitive high-grade ovarian carcinoma (ARIEL2 Part 1): an international, multicentre, open-label, phase 2 trial. Lancet Oncol. 2017;18(1):75-87. doi:10.1016/S1470-2045(16)30559-9 27908594

[25] Gomez-Puerto D, Llop-Guevara A, Cruellas M, . Genetic and functional homologous repair deficiency as biomarkers for platinum sensitivity in TNBC: a case report. Front Oncol. 2022;12:963728. doi:10.3389/fonc.2022.963728 36185283 PMC9516106

[26] Pujade-Lauraine E, Brown J, Barnicle A, . Homologous recombination repair gene mutations to predict olaparib plus bevacizumab efficacy in the first-line ovarian cancer PAOLA-1/ENGOT-ov25 trial. JCO Precis Oncol. 2023;7:e2200258. doi:10.1200/PO.22.00258 36716415 PMC9928987

[27] O’Malley DM, Oza AM, Lorusso D, . Clinical and molecular characteristics of ARIEL3 patients who derived exceptional benefit from rucaparib maintenance treatment for high-grade ovarian carcinoma. Gynecol Oncol. 2022;167(3):404-413. doi:10.1016/j.ygyno.2022.08.021 36273926 PMC10339359

[28] Bonnet E, Haddad V, Quesada S, . Alterations in homologous recombination-related genes and distinct platinum response in metastatic triple-negative breast cancers: a subgroup analysis of the ProfiLER-01 trial. J Pers Med. 2022;12(10):1595. doi:10.3390/jpm12101595 36294734 PMC9604780

[29] Richards S, Aziz N, Bale S, ; ACMG Laboratory Quality Assurance Committee. Standards and guidelines for the interpretation of sequence variants: a joint consensus recommendation of the American College of Medical Genetics and Genomics and the Association for Molecular Pathology. Genet Med. 2015;17(5):405-424. doi:10.1038/gim.2015.30 25741868 PMC4544753

[30] Denkert C, Romey M, Swedlund B, . Homologous recombination deficiency as an ovarian cancer biomarker in a real-world cohort: validation of decentralized genomic profiling. J Mol Diagn. 2022;24(12):1254-1263. doi:10.1016/j.jmoldx.2022.09.004 36191839

[31] Timms KM, Abkevich V, Hughes E, . Association of *BRCA1/2* defects with genomic scores predictive of DNA damage repair deficiency among breast cancer subtypes. Breast Cancer Res. 2014;16(6):475. doi:10.1186/s13058-014-0475-x 25475740 PMC4308910

[32] Loibl S, Weber KE, Timms KM, . Survival analysis of carboplatin added to an anthracycline/taxane–based neoadjuvant chemotherapy and HRD score as predictor of response-final results from GeparSixto. Ann Oncol. 2018;29(12):2341-2347. doi:10.1093/annonc/mdy460 30335131

[33] Telli ML, Timms KM, Reid J, . Homologous recombination deficiency (HRD) score predicts response to platinum-containing neoadjuvant chemotherapy in patients with triple-negative breast cancer. Clin Cancer Res. 2016;22(15):3764-3773. doi:10.1158/1078-0432.CCR-15-2477 26957554 PMC6773427

[34] Boni J, Idani A, Roca C, . A decade of *RAD51C* and *RAD51D* germline variants in cancer. Hum Mutat. 2022;43(3):285-298. doi:10.1002/humu.24319 34923718

[35] Kondrashova O, Nguyen M, Shield-Artin K, ; AOCS Study Group. Secondary somatic mutations restoring *RAD51C* and *RAD51D* associated with acquired resistance to the PARP inhibitor rucaparib in high-grade ovarian carcinoma. Cancer Discov. 2017;7(9):984-998. doi:10.1158/2159-8290.CD-17-0419 28588062 PMC5612362

[36] Kahn RM, Selenica P, Boerner T, . Pathogenic germline variants in non-*BRCA1/2* homologous recombination genes in ovarian cancer: analysis of tumor phenotype and survival. Gynecol Oncol. 2024;180:35-43. doi:10.1016/j.ygyno.2023.11.019 38041901 PMC10922242

[37] Srinivasan P, Bandlamudi C, Jonsson P, . The context-specific role of germline pathogenicity in tumorigenesis. Nat Genet. 2021;53(11):1577-1585. doi:10.1038/s41588-021-00949-1 34741162 PMC8957388

[38] Chopra N, Tovey H, Pearson A, . Homologous recombination DNA repair deficiency and PARP inhibition activity in primary triple negative breast cancer. Nat Commun. 2020;11(1):2662. doi:10.1038/s41467-020-16142-7 32471999 PMC7260192

