# Supplementary material for: Prevalence of Homologous Recombination Deficiency Among Patients With Germline RAD51C/D Breast or Ovarian Cancer
Source: JAMA Netw Open. 2024 Apr 22;7(4):e247811. doi: 10.1001/jamanetworkopen.2024.7811 (PMC11036141; doi:10.1001/jamanetworkopen.2024.7811)
Supplement: Supplement 1. — eTable 1. Unique Pathogenic Variants in RAD51C (n=56) eTable 2. Unique Pathogenic Variants in RAD51D (n=35) eFigure 1. CONSORT Diagram eFigure 2. Analysis of Functional HRD Biomarkers by Immunofluorescence eFigure 3. Distribution of Functional HRD Across Tumors With Pathogenic Variants in RAD51C/D eFigure 4. Concordance Between HRD Tests: Functional HRD by RAD51, Genomic HRD by GIS and RAD51C/D Gene-Specific LOH eFigure 5. Comparison of HRR/gsLOH Status With Age at Diagnosis and Cancer Subtype [file jamanetwopen-e247811-s001.pdf]

Supplementary Online Content

Torres-Esquius S, Llop-Guevara A, Gutiérrez-Enríquez S, et al. Prevalence of homologous recombination deficiency among patients with germline *RAD51C/D* breast or ovarian cancer. *JAMA Netw Open.* 2024;7(4):e247811. doi:10.1001/jamanetworkopen.2024.7811

eTable 1. Unique Pathogenic Variants in *RAD51C* (n=56)

eTable 2. Unique Pathogenic Variants in *RAD51D* (n=35)

eFigure 1. CONSORT Diagram

eFigure 2. Analysis of Functional HRD Biomarkers by Immunofluorescence

eFigure 3. Distribution of Functional HRD Across Tumors With Pathogenic Variants in *RAD51C/D*

eFigure 4. Concordance Between HRD Tests: Functional HRD by RAD51, Genomic HRD by GIS and RAD51C/D Gene-Specific LOH

eFigure 5. Comparison of HRR/gsLOH Status With Age at Diagnosis and Cancer Subtype

This supplementary material has been provided by the authors to give readers additional information about their work.

© 2024 Torres-Esquius S et al. *JAMA Network Open.*

eTable 1. Unique Pathogenic Variants in *RAD51C* (n=56)

**Coding change**[‡]	**Protein change**[†]	**n (%)**
c.1026+5_1026+7del	p.? / (p.Arg322Serfs*22)[a]	11 (20)
c.709C>T	p.(Arg237*)	9 (16)
c.965+5G>A	p.(Glu303Trpfs*41)	5 (9)
Deletion exons 4 to 5	p.?	4 (7)
c.404G>A	p.(Cys135Tyr) / p.?	4 (7)
c.358dupA	p.(Thr120fs)	2 (4)
c.577C>T	p.(Arg193*)	2 (4)
c.656T>C	p.(Leu219Ser)	2 (4)
c.705+1G>A	p.?	2 (4)
c.706-2A>G	p.? / p.(Arg237_Val280del) [a]	2 (4)
c.904+5G>T	p.? / p(Val280Glyfs*11)[b]	2 (4)
c.934C>T	p.(Arg312Trp)	2 (4)
Deletion exons 4 to 9	p.?	2 (4)
c. 405_571del	p.(Cys135*)	1 (2)
c.571+2T>A	p.?	1 (2)
c.706-1G>A	p.?	1 (2)
c.732delT	p.(Ile244Metfs*9)	1 (2)
c.890_899del	p.(Leu297Hisfs*2)	1 (2)
c.905-2_905-1del	p.? / p.(Leu301Gglyfs*42)[c]	1 (2)
c.955C>T	p.(Arg319*)	1 (2)
c.979_989dup	p.(Ser331Thrfs*37)	1 (2)

[‡]NM_058216.3

[†] NP 478123.1

[a] Truncating protein effect predicted in: G. Davy et al., EJHG 2017 (PMID: 28905878)

[b] Truncating protein effect predicted in: A. Meindl et al., Nat Genet 2010 (PMID: 20400964)

[c] Truncating protein effect predicted in: F. Lhota et al., Clin Genet 2016 (PMID: 26822949)

© 2024 Torres-Esquius S et al. *JAMA Network Open.*

eTable 2. Unique Pathogenic Variants in *RAD51D* (n=35)

**Coding change**[‡]	**Protein change**[†]	**n (%)**
c.694C>T	p.(Arg232*)	20 (56)
c.94_95delGT	p.(Val32fs)	5 (14)
c.898C>T	p.(Arg300*)	4 (11)
c.655C>T	p.(Gln219*)	3[§] (8)
c.620C>T	p.(Ser207Leu)	1 (3)
c.649_655delinsTGAGGTT	p.(Gly217_Gln219delins*)	1 (3)
c.649G>T	p.(Gly217*)	1[§] (3)
c.748del	p.(His250fs*)	1 (3)

[‡] NM_002878.3

[†] NP_002869.3

[§] one participant carries two PV in *RAD51D* c.655C>T and c.649G>T

© 2024 Torres-Esquius S et al. *JAMA Network Open.*

eFigure 1. CONSORT Diagram**.** A total of 181 patients with germline *RAD51C/D* pathogenic mutations were included in this study. Untreated BC/OC samples were obtained from 45 patients and 22 tumor samples were evaluable for both RAD51 and GIS.

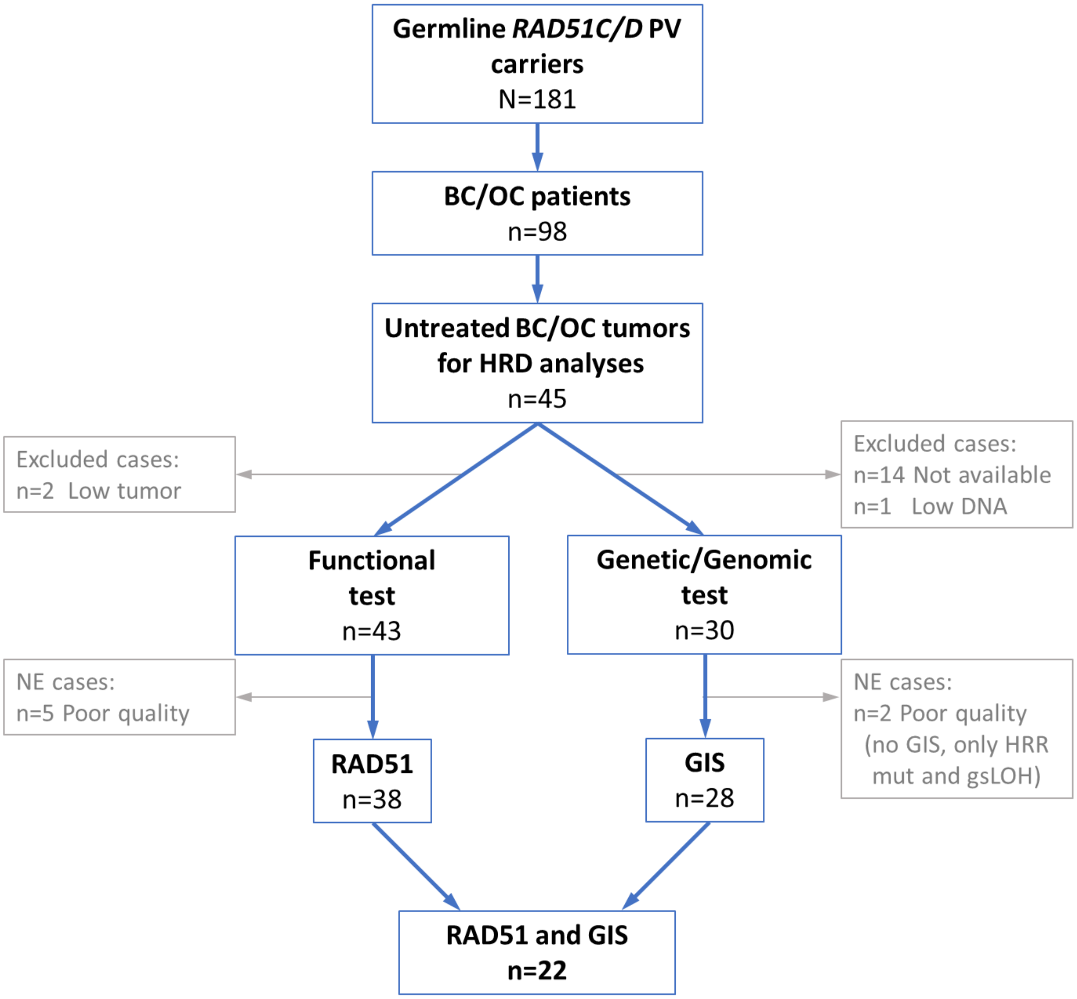

© 2024 Torres-Esquius S et al. *JAMA Network Open.*

eFigure 2. Analysis of Functional HRD Biomarkers by Immunofluorescence. Percentages of geminin-positive tumor cells with nuclear foci of yH2AX (marker of dsDNA damage), *BRCA1* and RAD51 in 38 samples. The line indicates the mean score of each biomarker and the dotted lines show the pre-defined thresholds to discriminate biomarker high vs low tumors.

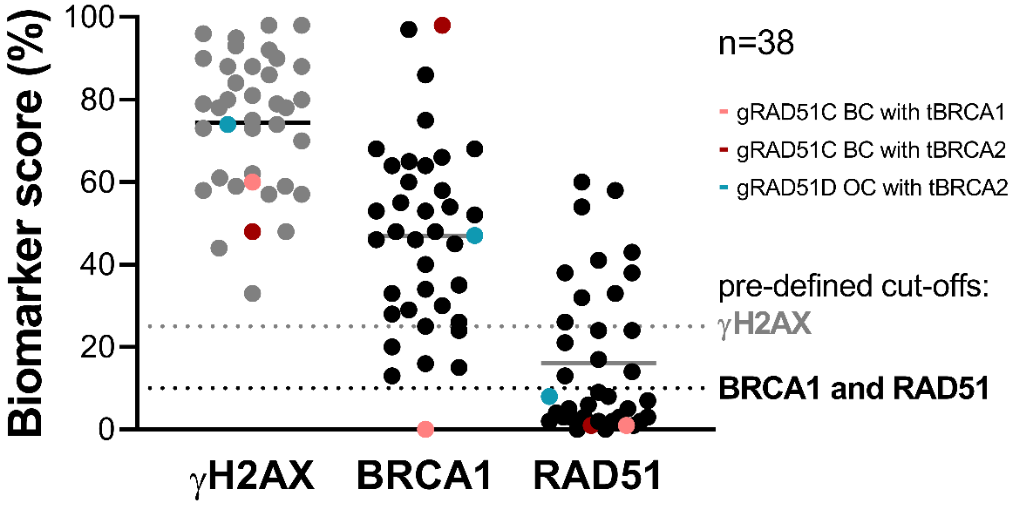

© 2024 Torres-Esquius S et al. *JAMA Network Open.*

eFigure 3. Distribution of Functional HRD Across Tumors With Pathogenic Variants in *RAD51C/D*. **A)** RAD51 scores in 29 tumors with distinct mutations in *RAD51C*. **B)** RAD51 scores in 9 tumors with distinct mutations in *RAD51D*. Each dot represents one tumor per patient. The dotted line at 10% is the threshold of the RAD51 test to discriminate HRD vs HRP tumors.

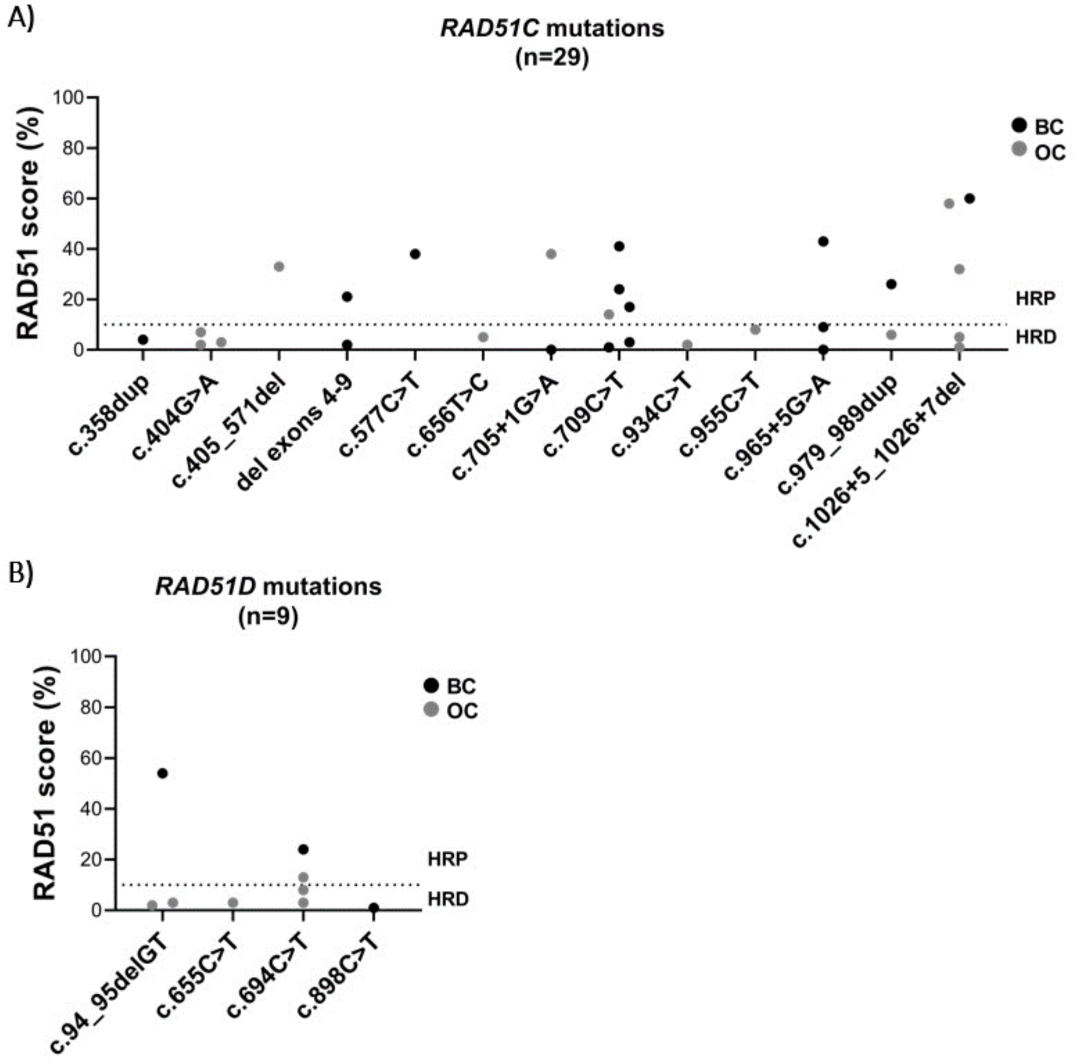

© 2024 Torres-Esquius S et al. *JAMA Network Open.*

eFigure 4. Concordance Between HRD Tests: Functional HRD by RAD51, Genomic HRD by GIS and RAD51C/D Gene-Specific LOH. Number of tumors and concordance statistics for: **A)** RAD51 vs GIS, **B)** RAD51 vs gsLOH, and **C)** GIS vs gsLOH.

		**GIS**			
		**High (HRD)**	**Low (HRP)**	**Concordance**	**Cohen's κ**
**RAD51**	**Low (HRD)**	14	0	**91%**	0.79 (95%CI 0.52 - 1.00)
	**High (HRP)**	2	6		

		**gsLOH**			
		**yes**	**no**	**Concordance**	**Cohen's κ**
**RAD51**	**Low (HRD)**	15	0	**83%**	0.61 (95%CI 0.29 - 0.93)
	**High (HRP)**	4	5		

		**gsLOH**			
		**yes**	**no**	**Concordance**	**Cohen's κ**
**GIS**	**High (HRD)**	20	0	**76%**	0.43 (95%CI 0.15 - 0.71)
	**Low (HRP)**	8	g		

© 2024 Torres-Esquius S et al. *JAMA Network Open.*

eFigure 5. Comparison of HRR/gsLOH Status With Age at Diagnosis and Cancer Subtype. **A and B)** Distribution of RAD51 scores in breast and ovarian tumors by age of the patient at cancer diagnosis. Graphs also include information of the gsLOH status of each tumor. **C)** Association of RAD51 score or gsLOH status with age at diagnosis based on univariate logistic regression analysis. **D)** Number of tumors for each subtype with HRD or HRP based on RAD51 and GIS, as well as the gsLOH status. This graph summarizes the data of the table in **E**. (*) Two HGOC with gsLOH have been included as HRP despite being HRP by RAD51 and HRD by GIS.

A)

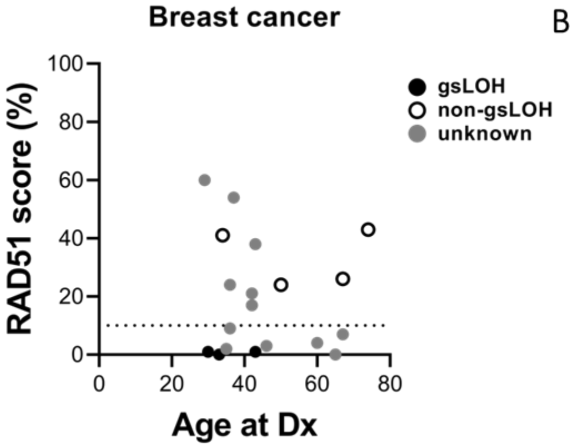

B)

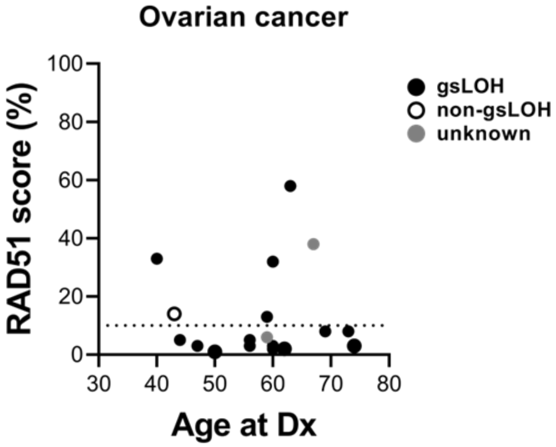

C)

**Association** with age at diagnosis	**OC**	**BC**	**OC + BC**
RAD51 score	$p = 0.39$	$p = 0.91$	$p = 0.27$
gsLOH status	$p = 0.31$	$p = 0.13$	$p = 0.89$

D)

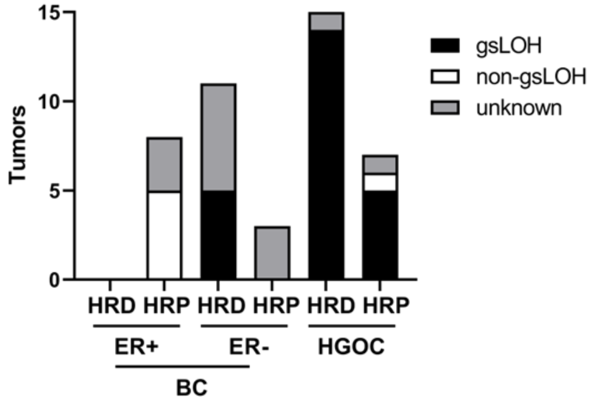

E)

Cancer subtype	HRR status	gsLOH	non-gsLOH	unknown	Total
ER+ BC	HRD	0	0	0	**0**
	HRP	0	5	3	**8**
ER- BC	HRD	5	0	6	**11**
	HRP	0	0	3	**3**
HGOC	HRD	14	0	1	**15**
	HRP*	5	1	1	**7**
	Total	**24**	**6**	**14**	**44**

© 2024 Torres-Esquius S et al. *JAMA Network Open.*
