# Supplementary material for: Prevalence of Homologous Recombination Deficiency Among Patients With Germline RAD51C/D Breast or Ovarian Cancer
Source: JAMA Netw Open. 2024 Apr 22;7(4):e247811. doi: 10.1001/jamanetworkopen.2024.7811 (PMC11036141; doi:10.1001/jamanetworkopen.2024.7811)
Supplement: Supplement 2. — Data Sharing Statement [file jamanetwopen-e247811-s002.pdf]

Data Sharing Statement

Torres-Esquius. Prevalence of Homologous Recombination Deficiency Among Patients With Germline RAD51C/D Breast or Ovarian Cancer. *JAMA Netw Open*. Published April 22, 2024. doi:10.1001/jamanetworkopen.2024.7811

Data
Data available: No

Additional Information
Explanation for why data not available: Due to concerns regarding patient privacy, the datasets employed in this study are not accessible to the public; however, upon reasonable request to the corresponding author, they may be made accessible. Please contact Judith Balmana at jbalmana@vhio.net for additional details.
